# Real-World Data: Implementation and Outcomes of Next-Generation Sequencing in the MENA Region

**DOI:** 10.3390/diagnostics15101183

**Published:** 2025-05-08

**Authors:** Rami Mahfouz, Reine Abou Zeidane, Tasnim Diab, Ali Tarhini, Eman Sbaity, Houry Kazarian, Yomna El Zibaoui, Nour Sabiha Naji, Mounir Barake, Hazem I. Assi

**Affiliations:** 1 Department of Pathology and Laboratory Medicine, American University of Beirut Medical Center, Beirut P.O. Box 11-0236, Lebanon; rm11@aub.edu.lb; 2 Department of Internal Medicine, Division of Hematology and Oncology, American University of Beirut Medical Center, Naef K Bassile Cancer Institute, Beirut P.O. Box 11-0236, Lebanon; reine.abz@gmail.com (R.A.Z.); td19@aub.edu.lb (T.D.); hk87@aub.edu.lb (H.K.); mounir.97@hotmail.com (M.B.); 3 Faculty of Medicine, American University of Beirut, Beirut P.O. Box 11-0236, Lebanon; aht13@mail.aub.edu (A.T.); yge07@mail.aub.edu (Y.E.Z.); nnaji1@jh.edu (N.S.N.); 4 Department of General Surgery, American University of Beirut Medical Center, Beirut P.O. Box 11-0236, Lebanon; es25@aub.edu.lb

**Keywords:** sequencing, NGS, molecular profiling, targeted therapy, precision medicine, personalized medicine

## Abstract

**Background:** In the era of precision medicine, Next-Generation Sequencing (NGS) has emerged as an important tool for identifying targetable mutations and tailoring treatment options. Yet the Middle East and North Africa (MENA) lags behind in adopting this technology. This study aims to demonstrate the transformative potential of molecular profiling in the region. **Methods:** This retrospective study reviewed cancer patients at the American University of Beirut Medical Centre, comparing outcomes between those who received NGS-based treatment adjustments (NBTAs) and those who did not. **Results:** The study enrolled 180 patients, including those with non-small-cell lung cancer (21.2%), sarcomas (20%), gastrointestinal malignancies (23.3%), breast cancer (10.6%), and other cancers (24.9%); 58.3% had stage 4 cancer at diagnosis. Before molecular profiling, 20.6% had stable disease, 21.7% showed partial response, and 57.8% had progressive disease. Most (96%) had received treatment, mainly systemic (90%), with chemotherapy (89%) being the most common. Forty patients (22.2%) underwent NGS-based treatment adjustments (NBTAs). Post-NGS, targeted therapies increased from 35% to 43% and immunotherapies from 14% to 18%. Mutations were detected in 98% of patients, with a median of four mutations per patient. NBTA patients had a median overall survival of 59 months, compared to 23 months for non-NBTA patients (*p* = 0.096), and significantly improved progression-free survival (5.32 vs. 3.28 months, *p* = 0.023). **Conclusions:** The use of large-scale molecular profiling to guide treatment adjustments promises advancements in patient care. Integrating NGS into clinical practice correlates with improved PFS, calling for a broader adoption of its use in the MENA region.

## 1. Introduction

Precision oncology, which enables personalized treatment for cancer patients, has experienced significant growth in the last decade due to the expanding field of genome sequencing [1]. Specifically, advancements in Next-Generation Sequencing (NGS) techniques and its growing accessibility and affordability have made panel testing for hundreds of cancer-related genes possible. This testing helps identify tumor mutations and recommend specific targeted treatments [2]. NGS has broadened the spectrum of tumor profiling from single variations to molecular signatures like tumor mutational burden (TMB) and Microsatellite Instability (MSI). The latter has been regarded as a gamechanger in immunotherapy administration.

NGS paved the way for specific targeted therapies, allowing personalized patient treatment while offering the potential to circumvent chemotherapy and its adverse systemic effects. Due to its precision and specificity, targeted therapy has been proven in several studies to improve survival parameters while decreasing adverse effects when compared to chemotherapy [3,4].

The inherent resistance and the ability of cancer to acquire resistance to drugs, whether chemotherapeutic or targeted, pose a challenge to treatment selection and vary among different cancer types [5]. The advancements in NGS have aided in identifying the genomic mechanisms underlying multi-drug resistance in solid tumors and leukemias, as well as in determining the most appropriate treatment options based on the identified mutations [6,7].

Advancements in genome sequencing have introduced several NGS assays, including Targeted Gene Panels, which are the most commonly used and have the highest depth of coverage. This ranges from Whole-Exome Sequencing (WES) to Whole-Genome Sequencing (WGS), which is the most comprehensive, and RNA sequencing [8]. The preferred solid tumor specimens are usually fixed in formalin buffer and embedded in paraffin (FFPE) [9]. Another technique is the use of liquid biopsies to measure Circulating Tumor DNA (ctDNA) or Cell-Free DNA (cfDNA) in peripheral blood samples, which is a valid alternative in certain pathologies and in situations where tissue re-biopsy is not feasible [8].

However, despite significant progress in the NGS field, it still faces barriers related to costs and availability. These barriers include the high cost of analysis and infrastructure, cost disparities between low- and high-income countries, extended turnaround time for results, ethical considerations, and the fact that not all NGS outputs offer indications for targeted therapy. Moreover, the absence of established investment plans and health technology assessment frameworks in many MENA countries exacerbates these challenges, resulting in disparities in access to advanced genomic diagnostics and personalized cancer treatments [10]. In addition, expertise is needed in the level of analytical and technical performance (Informatics/Bioinformatics) required for the successful implementation of any NGS-based diagnostic service.

Moreover, despite the rapid advancement of NGS, its clinical applications remain without specific guidelines. Recently, certain oncology societies have begun establishing recommendations for integrating NGS-based treatments into clinical practice, including the Food and Drug Administration (FDA) [11], the European Society for Medical Oncology (ESMO) Precision Medicine Working Group (PMWG) [12], and the Korean Society of Medical Oncology (KSMO) [13]. Other societies, like the American Association for Molecular Pathology (AMP) and the College of American Pathologists (CAP), have taken their role in setting algorithms for testing and sample requirements, analysis protocols, and validation guidelines with proper quality control material and metrics for proper implementation.

Despite the recommendations, the clinical value of NGS is yet to receive a clear consensus. This is why there are many studies in the literature aiming to determine the effect of NGS-based therapy on survival parameters. A recent review by the American Society of Clinical Oncology (ASCO) examined the available evidence regarding the use of NGS in treatment outcomes in the United States. This comprehensive review included twenty-nine studies, with patient numbers ranging from 35 to 5688 [11]. However, exploring the clinical implications of NGS-based treatment adjustments on survival parameters remains surprisingly underexplored outside the US, particularly in the MENA region.

Due to the widespread use of NGS, it has become common in developed countries to genomically profile cancer patients early on in the course of management of these patients. However, the latest evidence indicates that the majority of patients undergo genomic profiling late in their disease course [14]. Further research is needed to utilize NGS in the early stages of cancer, as most current studies suggest that genomic sequencing is conducted in the advanced stages of disease after multiple treatment regimens have been unsuccessful [15].

In this study, we aim to identify cancer patients who underwent molecular profiling and had a positive targetable mutation, assess the use of NGS-targeted treatment, and examine its impact on survival outcomes. This study is the first of its kind in Lebanon and across the MENA region, providing real-world national evidence from a tertiary healthcare center. It is our hope that this study will serve as the initial phase towards a comprehensive series of trials in the Middle East, which can then be used to establish clear guidelines and recommendations.

## 2. Materials and Methods

This retrospective real-world chart review study analyzed data from patients’ medical and electronic health records at the American University of Beirut Medical Center (AUBMC), a tertiary healthcare and major referral center in Lebanon. The study included patients aged 18 or older with advanced (stages III or IV), metastatic, refractory, or recurrent cancer who underwent molecular profiling between January 2016 and September 2022. All patients underwent NGS testing using Foundation Medicine and Guardant360, both of which follow established guidelines and algorithms for variant interpretation, ensuring standardized and clinically validated molecular profiling.

This study was conducted in accordance with the ethical principles outlined in the Declaration of Helsinki (2013). Approval was granted by the Institutional Review Board (BIO-2020-0569) at AUBMC prior to data collection, and a waiver of consent was obtained. All data were anonymized to ensure confidentiality, and the authors did not have access to any information that could identify individual participants during or after data collection.

Demographic information, type of NGS test received, tumor types, staging, lines of therapy received before NGS testing, and disease status both before and after the NGS tests were collected. Actionable mutations were defined as variants validated in peer-reviewed research for which targeted therapies were available.

Continuous variables were described as means ± standard deviations or median (interquartile range), while categorical variables were presented as frequencies and percentages. Survival analysis was performed using Kaplan–Meier curves to evaluate overall survival (OS) and progression-free survival (PFS). This analysis aimed to compare patients who received NGS-based treatment adjustment (NBTA) to those who did not, employing the log-rank test. PFS was defined as the period between the treatment modification based on molecular profiling test results and the subsequent progression.

Data were collected retrospectively and maintained using REDCap software (Research Electronic Data Capture) Version 13.7.24, and analysis was carried out using SPSS software version 29. A *p*-value of <0.05 was considered statistically significant.

## 3. Results

A total of 180 patients, with a median age of 57 years (range: 19–92), were included in our study. The majority of patients presented with advanced disease, with 58.3% having stage IV at the time of diagnosis. Prior to molecular profiling, 20.6% of patients had stable disease, 21.7% showed a partial response, and 57.8% had progressive disease. Molecular profiling was conducted at varying stages of the disease course; 21.1% of patients underwent early profiling, while the majority (78.9%) underwent late profiling (Table 1).

**Figure 1:**
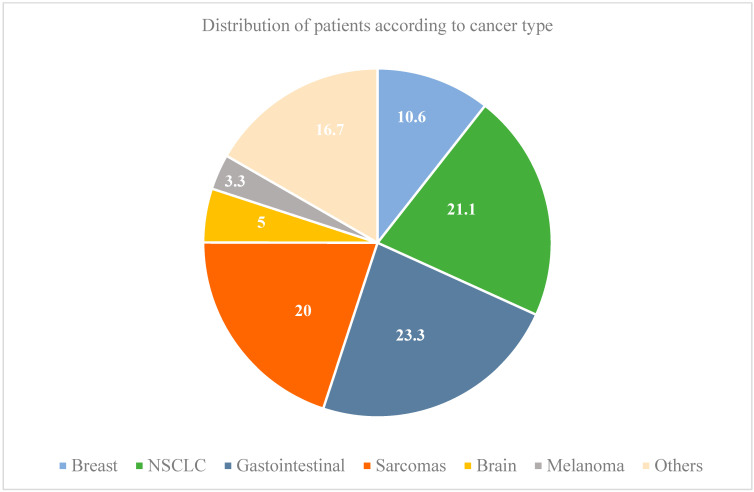
Distribution of patients according to cancer type.

A variety of molecular profiling tests were ordered at our institution, including FoundationOne CDx (67.8% of our patients), FoundationOne Heme (17.8%), FoundationOne Liquid (2.2%), FoundationOne Liquid CDx (8.3%), and Guardant 360 (3.9%) (Table 2). Molecular profiling was predominantly performed for patients diagnosed with non-small-cell lung cancer (NSCLC), soft tissue sarcoma, gastrointestinal malignancies, and breast cancer (Figure 1).

**Figure 2:**
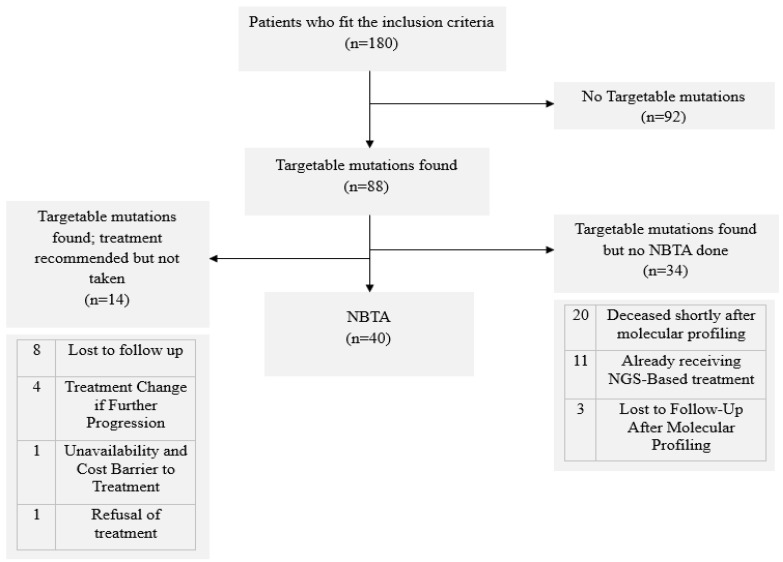
Molecular profiling-based treatment changes.

Out of the 180 patients who met the inclusion criteria, 88 were found to have targetable (actionable) mutations. Among these, 40 received NGS-based treatment adjustment (NBTA). For 34 patients, targetable mutations were identified, but no NBTA was performed due to patients dying shortly after profiling or being lost to follow-up. Additionally, 14 patients had targetable mutations and received treatment recommendations, but these were not pursued due to various reasons, including unavailability, cost barriers, and patient refusal, as detailed in Figure 2.

**Figure 3:**
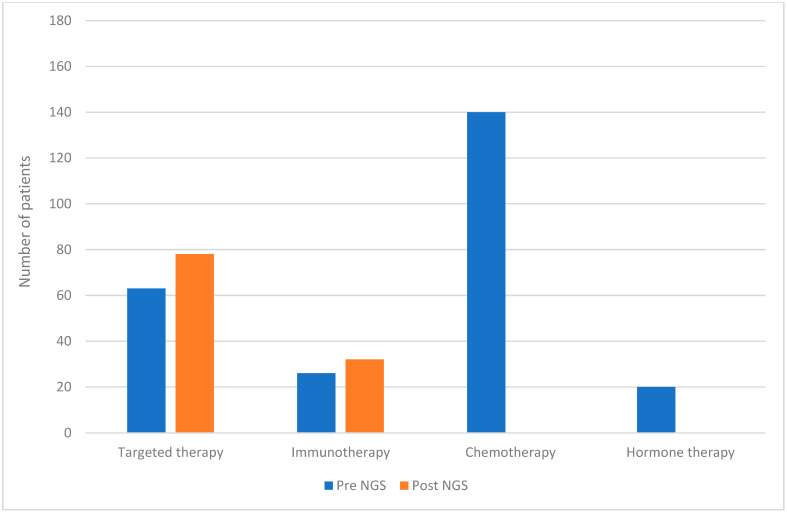
Proportion of patients who initiated immunotherapy, targeted therapy, or hormone therapy before or after NGS test results.

Prior to molecular profiling, systemic treatment was administered to 96% of patients, with chemotherapy being the most common systemic treatment (89%). Following NGS results, there was an increase in the proportion of patients receiving targeted therapies (from 35% to 43%) and immunotherapies (from 14% to 18%) (Figure 3).

**Figure 4:**
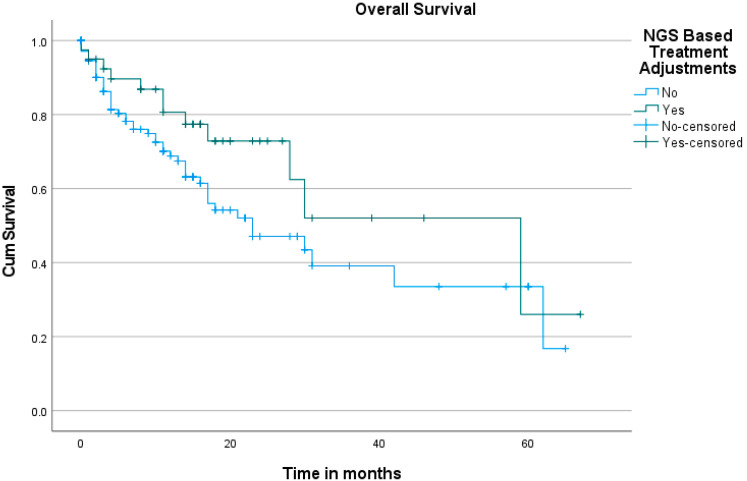
Kaplan–Meier curve of overall survival in patients receiving NBTA compared to those who did not.

**Figure 5:**
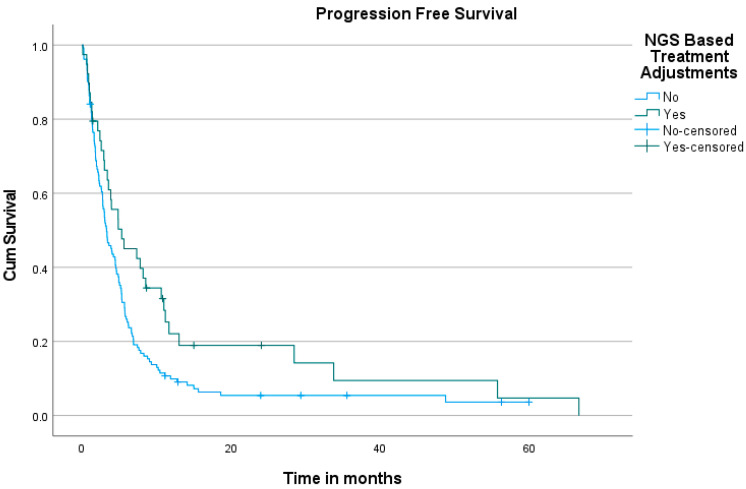
Kaplan–Meier curve of progression-free survival in patients receiving NBTA compared to those who did not.

Mutations were detected by NGS in 177 out of 180 patients, accounting for 98% of the study population. The median number of mutations found per patient was 4 (1–19). The median overall survival (OS) was 59 months (95% CI: 26.256 to 91.744 months) for those who underwent NBTA compared to 23 months (95% CI: 10.227 to 35.773 months) for those who did not (*p* = 0.096), with a 2-year OS of 73% versus 47%, respectively (Figure 4). The progression-free survival (PFS) difference was statistically significant (*p* = 0.023), with a median PFS of 5.32 months (95% CI: 1.268 to 9.384 months) for patients who received NBTA compared to 3.28 months (95% CI: 2.472 to 4.103 months) for non-NBTA patients (Figure 5).

Regarding early vs. late molecular profiling during the disease course in patients receiving NBTA, the median OS was 59 months for early profiling and 30 months for late profiling (*p* = 0.945). The median PFS was 10.9 months (95% CI: 3.145 to 18.816 months) for early profiling and 4.83 months (95% CI: 2.851 to 6.814 months) for late profiling (*p* = 0.520). These results suggest no statistically significant survival advantage based on the timing of molecular profiling in this subset of patients.

The median OS for patients who underwent NGS early in their treatment course was 30 months (95% CI: 3.878 to 56.122 months), which was similar to the 30 months (95% CI: 21.135 to 38.865 months) observed for those who underwent NGS testing later in their treatment course (*p*-value = 0.747). However, the median PFS differed significantly between the two groups: patients who underwent NGS early in their disease course had a median PFS of 6.214 months (95% CI: 4.664 to 7.763 months), compared to 3.288 months (95% CI: 2.587 to 3.989 months) for those who underwent NGS testing late (*p*-value = 0.033).

Following NBTA, stable disease was observed in 20% of patients, with partial response in 25% and progressive disease in 40%, and 15% were lost to follow-up. Among the 157 patients with TMB information on their NGS reports, 71% had low TMB (≤5), 27% had intermediate TMB (>5 and <20), 2% had high TMB (≥20 and <50), and one patient (0.6%) had extremely high TMB (≥50). TMB was not associated with survival outcomes (*p* = 0.570 for OS and *p* = 0.911 for PFS).

Table 3 presents an overview of the genes in which somatic mutations were most frequently detected in patients across various tumor types in our cohort. Supplementary Figures S1–S6 display the most common genes with somatic mutations, along with their respective prevalence and frequency.

## 4. Discussion

The findings presented in this paper are from a retrospective cohort analysis conducted at a tertiary referral center in the Middle East, aimed at evaluating survival outcomes in cancer patients treated with precision medicine. Our analysis showed that PFS was significantly greater in patients receiving NBTA compared to those on standard therapy, and that PFS was also greater in patients who underwent molecular profiling early in their disease course compared to those who did so later. However, there were no differences in OS based on either NBTA status or the timing of molecular profiling.

Our findings align with most of the literature. While some studies have shown no difference in PFS among those receiving NBTA [16], most research evidence supports a significant PFS benefit for patients who received matched therapy following profiling [17,18,19,20,21,22]. Notably, most of these studies included a similar number of patients to our cohort.

In contrast, even though patients receiving NBTA had better OS outcomes than those on standard treatment, the results did not reach statistical significance. However, most reports and trials demonstrate an OS advantage for NBTA [18,23,24,25,26,27]. Notably, most of these trials included substantially more patients than our cohort, while those that showed no statistical significance also had smaller sample sizes [20,28,29,30]. Furthermore, not all studies in the literature assess multiple cancer types; many examine the effect of NGS on a single cancer type or subset of cancers (e.g., gastrointestinal or gynecologic cancer). While this approach has its benefits, including patients with various cancer types enhances generalizability and real-world applicability.

The most common somatic variants detected using NGS in the literature included TP53, CDK4, MDM2, RB1, and CDKN2A/B for sarcomas [31], ERBB2, PIK3CA, and ESR1 for breast cancer, EGFR and KRAS for NSCLC, KIT and IDH1/2 for gastrointestinal tumors [12], and TP53, IDH1, PIK3CA, PTEN, and EGFR for brain tumors [32]. Some of those findings show similarities to our cohort, but there are also differences in the most prevalent variants, likely due to the different populations and ethnicities. This allows for tailoring future studies to target specific ethnic groups, allowing for even more tailored management and guidelines.

Most studies previously referenced are U.S.-based, with other international studies showing similar outcomes. A study in France evaluating NGS across multiple cancer types found that 8% of participants received targeted therapy post-genomic profiling (a 3% increase from pre-profiling) and demonstrated improved one-year survival compared to those without NGS-guided therapy [33]. Additional studies in France [34], China (on esophageal cancer patients) [35], and Japan (on cancer of unknown primary) [36] have all shown favorable OS or PFS outcomes. In contrast, the SHIVA trial, a French randomized controlled trial, showed no benefit of NBTA on PFS [37]. This trial had several limitations, including using a predefined algorithm for matched therapy versus the physician’s choice of treatment for controls and limiting patients to monotherapy instead of combination therapy [38,39]. Other studies found promise in NGS-based target therapy for several cancer types including sarcomas [31,40,41], melanomas [42], and gastrointestinal tumors [43,44], similar to our findings.

Nonetheless, the robust evidence for survival advantage paved the way for the FDA to approve over 28 different targeted treatments based on NGS [11]. Furthermore, the European Society for Medical Oncology (ESMO) Precision Medicine Working Group (PMWG) now recommends NGS for non-squamous non-small-cell lung cancer (NSCLC), ovarian cancer, prostate cancer, colorectal cancer, cholangiocarcinoma, advanced breast cancer, sarcomas, thyroid cancer, and gastrointestinal stromal tumors [12]. The Korean Society for Medical Oncology (KSMO) also endorses NGS testing for patients with metastatic or advanced solid tumors, even in cancers with rare actionable genetic alterations [13].

Despite its advantages, precision medicine faces limitations. NGS testing requires a growing array of tests, and the proportion of patients eligible for targeted therapies remains limited. Even when driver targets are identified, available drugs may not exist [45], or patients may face accessibility challenges, such as high costs and limited insurance coverage. Other limitations include analytic sensitivity of mutation detection, error rates, complex genomic regions to analyze, and data storage [46,47]. Precision medicine also raises ethical concerns, such as informed consent, data protection, and management of incidental germline findings [48,49].

In the MENA region, financial and infrastructural limitations restrict precision medicine adoption. Low healthcare spending, lack of insurance, and high NGS costs mean that NGS testing remains individual-based rather than population-based, limiting access to those who can afford it [10]. Furthermore, the MENA region lacks facilities providing NGS services, with only 16 laboratories accredited by the College of American Pathologists (CAP) for molecular testing. Consequently, MENA countries rely on facilities in the U.S. and Europe for NGS testing [10,50]. Additionally, only three MENA countries participate in Genome-Wide Association Studies (GWASs), collaborating with non-MENA countries to conduct these studies. Still, those countries collaborated with non-MENA countries to perform the aforementioned studies [51]. Moreover, even where NGS is available, MENA facilities face shortages in specialized equipment, trained personnel, expertise, and prolonged testing times [14,15], which challenges the routine, population-wide implementation of NGS testing [52,53].

To address these issues, the region should collaborate on making NGS more affordable, enhancing insurance coverage, training healthcare professionals in precision medicine, and increasing funding for infrastructure and facilities. Such efforts would support the integration of genomic testing into routine cancer care and enable patients to benefit from targeted treatments earlier. Our study demonstrated promising survival benefits of early NGS testing, and no studies, to our knowledge, specifically examine early versus late NGS testing on survival outcomes. Further investigation could clarify the clinical advantages of early NGS testing within the region, emphasizing the value of investing in molecular profiling. Further advances in NGS-based treatment can help expand on this work and help guide trials for combinations of immunotherapy and target therapy suggested by NGS with current standard treatments [54,55].

Our study has limitations. First, it is not a randomized controlled trial (RCT), which is the optimal study type for robust conclusions; however, our findings still suggest that molecularly guided therapies may improve survival in patients with advanced refractory cancer. Second, our data are limited to a single center with a small sample size. While most similar studies have comparable sample sizes and are monocentric, multi-center studies with larger samples would yield more robust and generalizable conclusions.

## 5. Conclusions

Many novel therapies require biomarkers. Unfortunately, a considerable percentage of patients in the MENA region who are eligible for molecular profiling tests do not undergo genetic testing due to commercial unavailability or high costs, even when testing is available. Furthermore, all commercially accessible molecular profiling assays have limited panels, each excluding at least one important biomarker recommended in clinical guidelines. Sequential molecular testing also has its disadvantages, potentially delaying treatment and leading to tissue exhaustion and depletion from excessive testing.

These obstacles need to be addressed in the MENA region to ensure that cancer patients can access alternative treatments. More research with larger sample sizes and prospective designs is needed to better understand the impact of molecular profiling and explore new personalized treatment approaches, considering potential side effects of targeted therapies. Innovative clinical trial designs that enhance the effectiveness of genetic testing and comprehensive data sharing are essential to maximize clinical benefits for all patients.

## Figures and Tables

**Table 1:** Demographics and clinical characteristics of patients enrolled.

	Patients *N* = 180
**Median age**	57 (19–92)
**Gender**	
Female	80 (44.4)
Male	100 (55.6)
**Cancer stage at diagnosis**	
I	18 (10)
II	16 (8.9)
III	30 (16.7)
IV	105 (58.3)
Not reported	11 (6.1)
**Cancer status prior to molecular profiling**	
Stable disease	37 (20.6)
Partial response	39 (21.7)
Progressive disease	104 (57.8)
**Molecular profiling in disease course**	
Early	38 (21.1)
Late *	142 (78.9)

Data are n (%) unless stated otherwise. * Cutoff = 2 months.

**Table 2:** Frequency of NGS test usage across malignancy types.

Malignancy	FoundationOne CDx	FoundationOne Heme	FoundationOne Liquid	FoundationOne Liquid CDx	Guardant 360
Sarcomas	3	31	1	0	1
Brain	9	0	0	0	0
Breast	17	0	1	0	1
Gastrointestinal	34	0	1	4	3
Melanoma	5	0	0	1	0
NSCLC	29	1	1	5	2
Others	25	0	0	5	0

**Table 3:** Genes in which somatic mutations were most frequently detected in patients.

	Breast	NSCLC	GI	Brain	Sarcomas	Others
Genes detected by NGS	n = 19	n = 38	n = 42	n = 9	n = 36	n = 30
Most commonly detected genes	TP53	TP53	KRAS	TERT	TP53	TP53
	PIK3CA	NF1/2	APC	TP53	CDK4	CDKN2A/B
	ZNF	KRAS	TP53	PTEN	MDM2	CCNE1
	AKT	KEAP1	NRAS	CDKN2A/B	FRS2	KRAS
	NSD3	STK11	PIK3CA	NF1	ATRX	FGF
	MDM2	ERBB2	FGF	ATRX	CDKN2A/B	TERT
	RAD21	CDKN2A/B	CDKN2A/B	EGFR	RB1	MYC
	GATA3	MET	FGF	MTAP	TET2	BRAF
	MYC	EGFR	SMAD4	IDH1	EWSR1	PIK3R1

## Data Availability

The original contributions presented in this study are included in the article/Supplementary Material. Further inquiries can be directed to the corresponding author(s).

## Supplementary Materials

The following supporting information can be downloaded at: https://www.mdpi.com/article/10.3390/diagnostics15101183/s1, Figure S1: Somatic Mutations in Breast Cancer. Figure S2: Somatic Mutations in NSCLC, Figure S3: Somatic Mutations in Gastrointestinal Malignancies, Figure S4: Somatic Mutations in Soft Tissue Sarcoma, Figure S5: Somatic Mutations in Bone Sarcoma, Figure S6: Somatic Mutations in Brain Tumors.
