# Supplementary material for: Real-World Data: Implementation and Outcomes of Next-Generation Sequencing in the MENA Region
Source: Diagnostics (Basel). 2025 May 8;15(10):1183. doi: 10.3390/diagnostics15101183 (PMC12110402; doi:10.3390/diagnostics15101183)

Supplementary Figure S1: Somatic Mutations in Breast Cancer

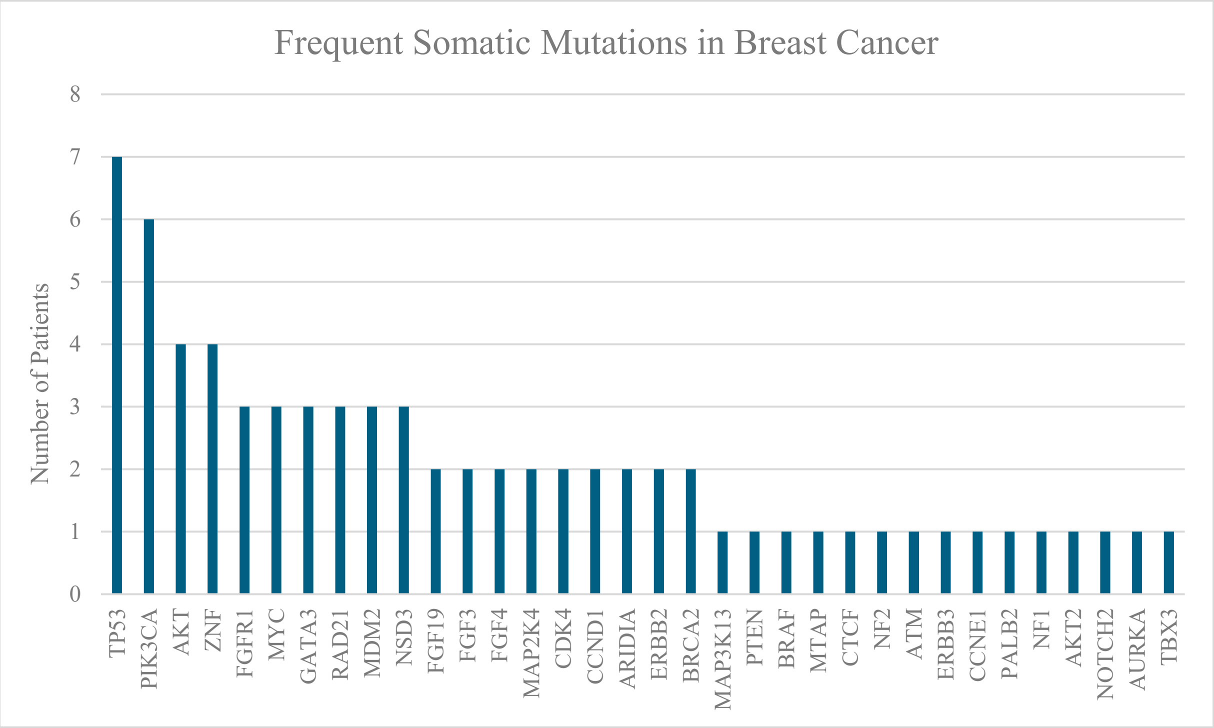

Supplementary Figure S2: Somatic Mutations in NSCLC

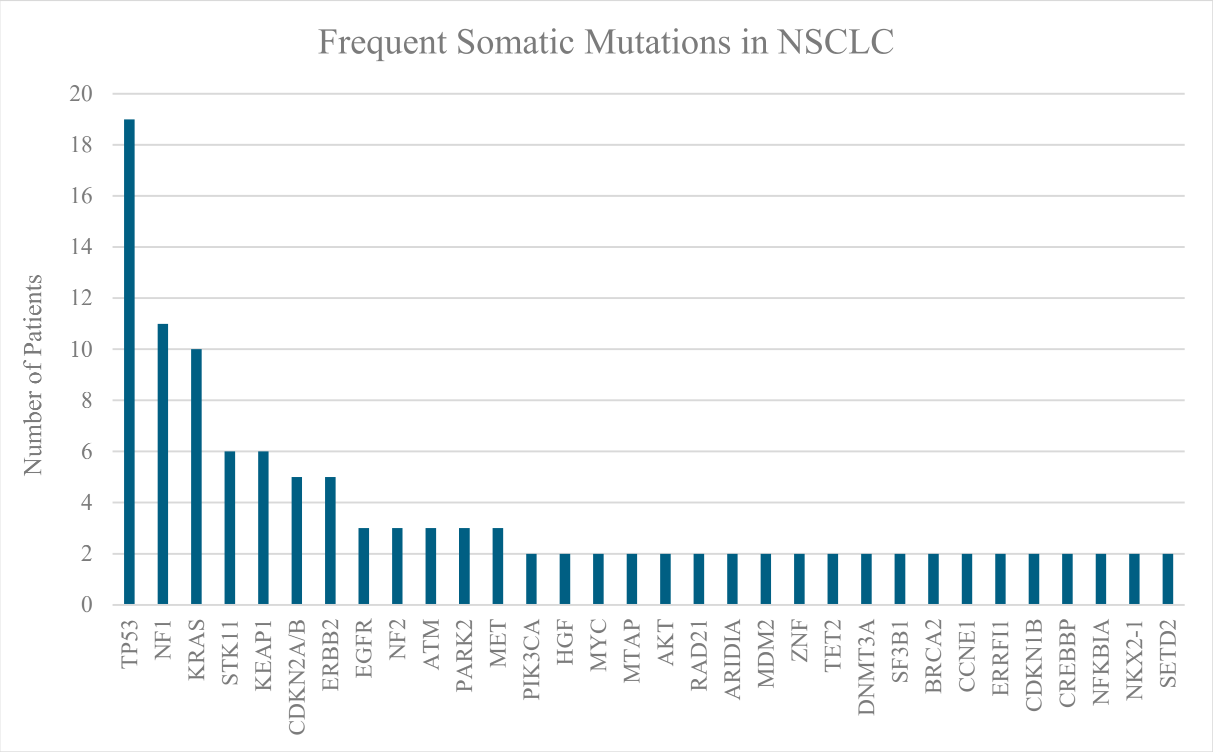

Supplementary Figure S3: Somatic Mutations in Gastrointestinal Malignancies

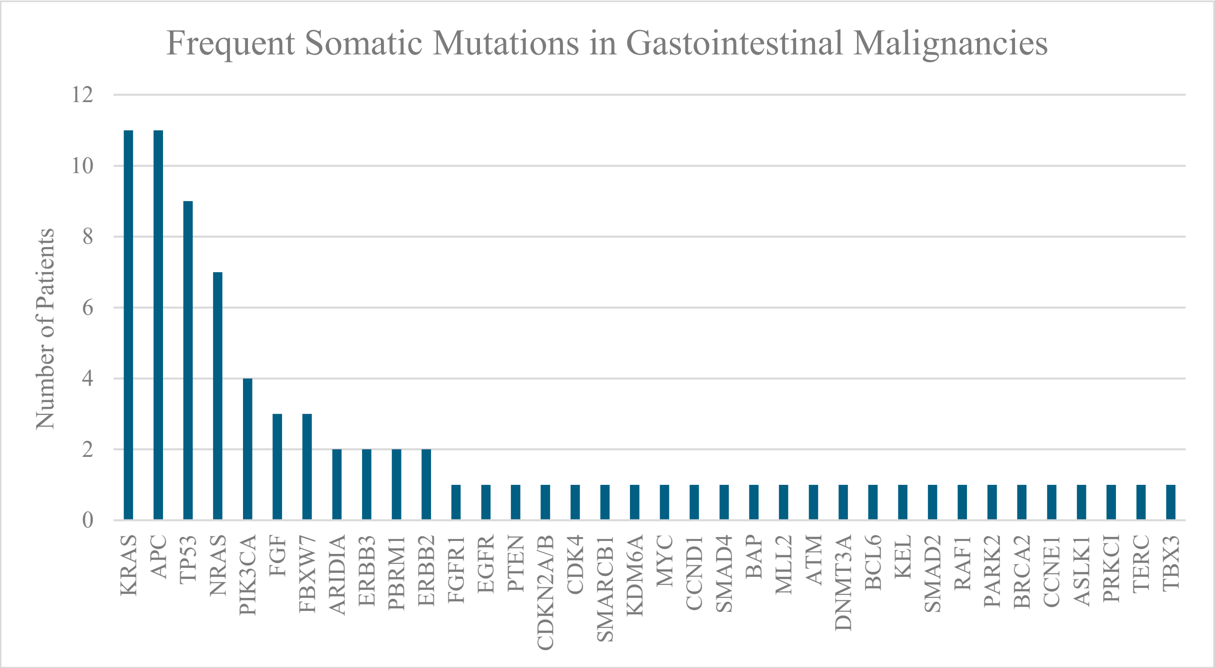

Supplementary Figure S4: Somatic Mutations in Soft Tissue Sarcoma

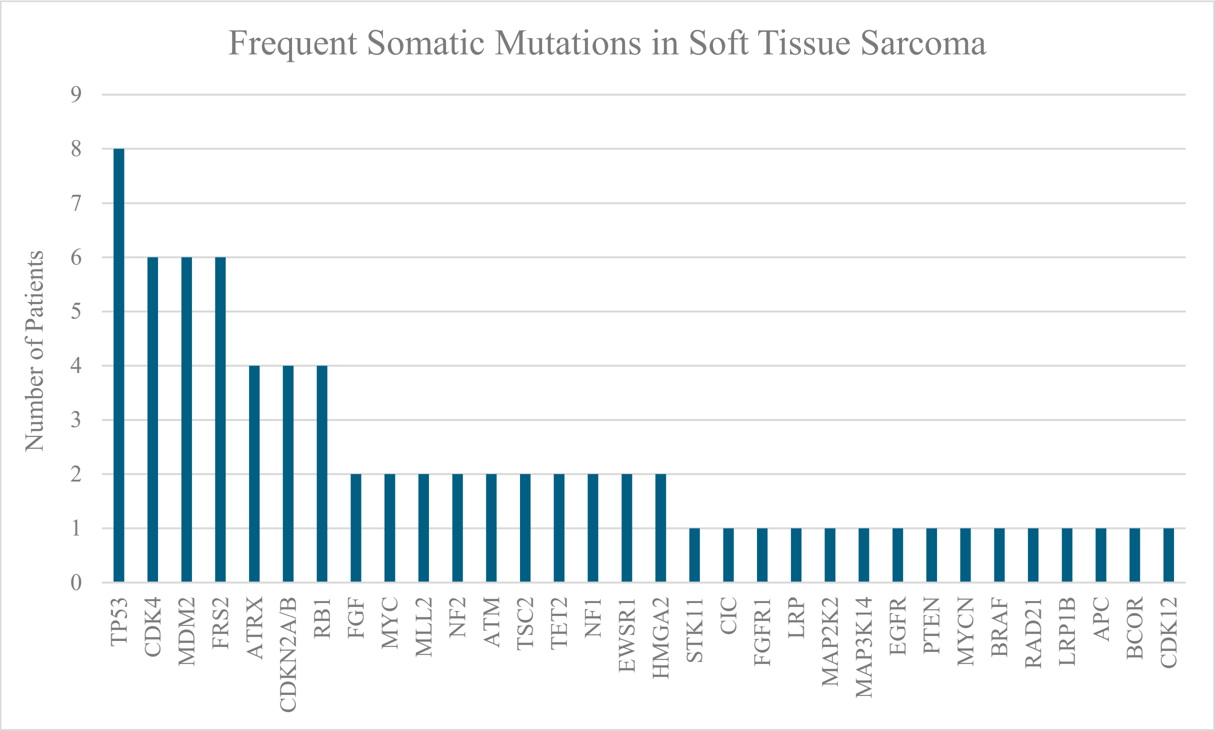

Supplementary Figure S5: Somatic Mutations in Bone Sarcoma

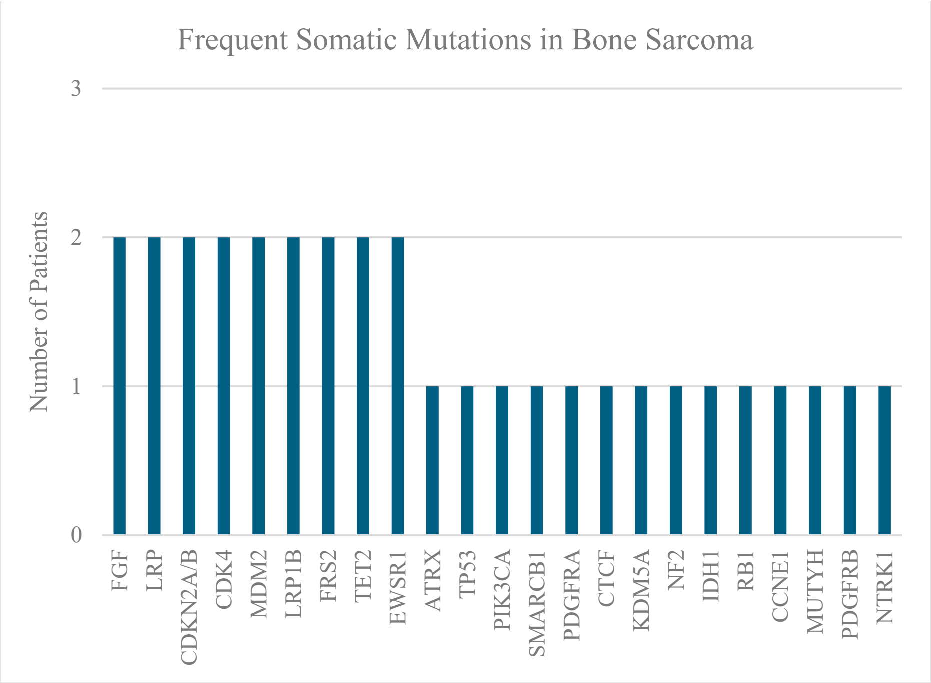

Supplementary Figure S6: Somatic Mutations in Brain Tumors

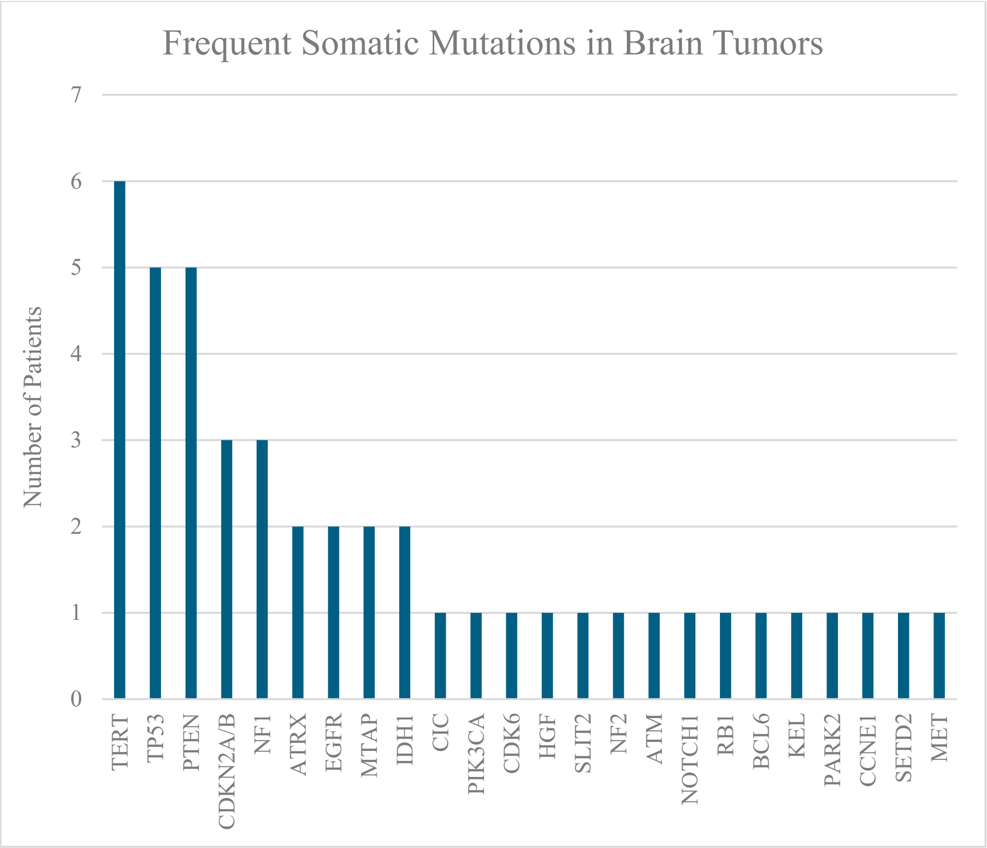

Supplement: Supplementary file 1 [file diagnostics-15-01183-s001.zip › diagnostics-3433894-supplementary.pdf]
